# Money Making

**Published:** 1870-07

**Authors:** 


### MONEY MAKING.

Many a man who has been complaining for months of being sick, and has fallen into laziness and discouragement, would get well in a week, if he was only put in the way of making money largely as to him, that is, five dollars a day to one who never earned but two; and a hundred to another who never earned but ten. Then again, it is an additional element of success, if there is an intelligent or scientific interest in the method.

A suggestion is therefore made which may revolutionize the commerce of nations, in the meanwhile making the fortunes of multitudes of individuals. All that is needed to make a beginning is two dollars and two yards of land ; the money will get several sprigs of

"THE RAMIE PLANT,"

by sending it in a post-office order, addressed to the Agricultural Bureau, Washington, D. C. It flourishes in California, Pennsylvania, and in other States; hence as a matter of scientific interest, why may not multitudes of invalids go into the business on the faith of the following statement of a Californian, confirmed in part by P. R. Freds, editor of the "Germantown Telegraph," one of the very best weekly newspapers in the United States, as its editor is one of the best of men.

"The ramie plant in the southern counties of California has passed beyond the range of an experiment. At the late Mechanics' Institute Fair, several samples of the raw material and its manufactured products, were exhibited. The report just published refers to it in the following manner: 'It yields a finer fibre than sea-island cotton, is stronger than the best flax or hemp, and is almost as brilliant as silk. It requires less labor to cultivate than cotton, is not destroyed by worms, does not suffer from excess of rain, and stands the longest drought without injury. It propagates rapidly, yields largely and commands a ready market at high prices.'"

